# PSAEEGNet: pyramid squeeze attention mechanism-based CNN for single-trial EEG classification in RSVP task

**DOI:** 10.3389/fnhum.2024.1385360

**Published:** 2024-05-02

**Authors:** Zijian Yuan, Qian Zhou, Baozeng Wang, Qi Zhang, Yang Yang, Yuwei Zhao, Yong Guo, Jin Zhou, Changyong Wang

**Affiliations:** ^1^School of Intelligent Medicine and Biotechnology, Guilin Medical University, Guangxi, China; ^2^Beijing Institute of Basic Medical Sciences, Beijing, China; ^3^Chinese Institute for Brain Research, Beijing, China

**Keywords:** P300, single-trial EEG, target recognition, convolutional neural network, rapid serial visual presentation, pyramid squeeze attention mechanism

## Abstract

**Introduction:**

Accurate classification of single-trial electroencephalogram (EEG) is crucial for EEG-based target image recognition in rapid serial visual presentation (RSVP) tasks. P300 is an important component of a single-trial EEG for RSVP tasks. However, single-trial EEG are usually characterized by low signal-to-noise ratio and limited sample sizes.

**Methods:**

Given these challenges, it is necessary to optimize existing convolutional neural networks (CNNs) to improve the performance of P300 classification. The proposed CNN model called PSAEEGNet, integrates standard convolutional layers, pyramid squeeze attention (PSA) modules, and deep convolutional layers. This approach arises the extraction of temporal and spatial features of the P300 to a finer granularity level.

**Results:**

Compared with several existing single-trial EEG classification methods for RSVP tasks, the proposed model shows significantly improved performance. The mean true positive rate for PSAEEGNet is 0.7949, and the mean area under the receiver operating characteristic curve (AUC) is 0.9341 (*p* < 0.05).

**Discussion:**

These results suggest that the proposed model effectively extracts features from both temporal and spatial dimensions of P300, leading to a more accurate classification of single-trial EEG during RSVP tasks. Therefore, this model has the potential to significantly enhance the performance of target recognition systems based on EEG, contributing to the advancement and practical implementation of target recognition in this field.

## 1 Introduction

Brain-computer interface (BCI) technology enables direct communication between humans and computers or other external devices by interpreting brain electrical activity (Cecotti and Graser, 2010; Manor and Geva, 2015). BCI technology has a wide range of applications across various domains, such as motion direction recognition (Zhang et al., 2022a), emotion recognition (Chen et al., 2019; Joshi and Ghongade, 2021; Tao et al., 2023), and epileptic seizure detection (Xu et al., 2020; Dissanayake et al., 2021; Jana and Mukherjee, 2021; Wang B. et al., 2023). Concurrently, researchers are actively investigating the potential application of electroencephalography (EEG) in the realm of target recognition (Lan et al., 2021). In complex environments, computer vision is prone to environmental disturbances, leading to diminished recognition capabilities. In contrast, human visual recognition has the problem of slow manual processing and low efficiency of human-computer interaction. Nevertheless, integrating human brain with computer vision can enhance both the accuracy and efficiency of target recognition in such intricate settings. Numerous studies have revealed that artificial intelligence (AI) methodologies effectively categorize event-related potentials (ERPs) produced by the brain of the subject when engaged in a rapid serial visual presentation (RSVP) task (Li et al., 2021; Tan et al., 2021; Zang et al., 2021). This classification process has considerable potential to improve EEG-based target recognition.

The RSVP paradigm is a common experimental approach for target recognition tasks. Within this paradigm, image stimuli are presented sequentially at a uniform frequency, typically ranging from 2 to 20 Hz, with each stimulus consistently occupying the same spatial location throughout the display (Zhang et al., 2020). When subjects successfully detect a target image in a rapid sequence, they excite P300, which is a specific component of the ERP (Lees et al., 2018). The RSVP paradigm is widely used in different scenarios, such as dim target detection in remote sensing images (Fan et al., 2022), dual-brain collaborative target detection (Zhang et al., 2021), and so on. The commonly used AI methods for processing single-trial EEG are deep learning and machine learning. In the past decades, researchers have proposed different algorithms based on traditional machine learning for single-trial EEG classification under RSVP tasks. The Common Sparse Spectral Spatial Pattern algorithm (Dornhege et al., 2006) is extended based on the Common Spatial Pattern algorithm for single-trial EEG analysis. Marathe et al. (2014) implemented a sliding window methodology in tandem with Hierarchical Discriminant Component Analysis to address temporal variability in neural responses, thereby enhancing the single-trial EEG classification accuracy. Alpert et al. (2013) introduced the Spatially Weighted FLD-PCA (SWFP) method, which is grounded on a two-stage linear classification approach for event-related responses. This method employs the Fisher Linear Discriminant classifier in conjunction with Principal Component Analysis to achieve dimensionality reduction, thereby enhancing the single-trial EEG classification performance. In the realm of single-trial EEG classification, conventional machine learning techniques often rely on linear algorithms, which are typically characterized by their swift training times and robustness. However, these methods may face limitations in terms of both the extraction of discriminative features and overall classification accuracy (Zang et al., 2021). This highlights the pressing requirement for new classification techniques that can more accurately and expediently extract meaningful features from single-trial EEG.

In recent years, deep learning techniques have been increasingly adopted for single-trial EEG classification tasks. As an end-to-end approach, deep learning obviates the need for manual feature design and extraction, instead leveraging its nonlinear computational prowess to derive features at multiple hierarchical levels. The CNN, a prevalent deep learning architecture, has proven particularly potent in image feature extraction. CNNs apply both temporal and spatial convolutions, thereby enabling them to extract crucial temporal and spatial characteristics from EEG with efficiency and precision (Schirrmeister et al., 2017; Zang et al., 2021). As a result, CNNs have become a popular choice for the analysis of EEG. For instance, Cecotti and Graser (2010) utilized CNN to extract spatial and temporal features from P300. Lawhern et al. (2018) proposed EEGNet, a compact CNN that employs a combination of deep and separable convolutions for more efficient feature extraction. Bhandari et al. (2023) have designed a novel, compact CNN architecture that integrates temporal dilated convolutions along with channel-wise attention mechanisms. This design aims to enhance the classification efficiency of P300. Macías-Macías et al. (2022) introduced a capsule neural network that has shown promising P300 classification performance with small samples and few channels. Furthermore, Wang Z. et al. (2023) proposed a novel method that integrates an attention module with a capsule neural network, aiming to enhance P300 classification effectiveness. Zhang et al. (2022b) proposed an improved EEGNet model, which enhances the signal-to-noise ratio through xDAWN filtering and addresses sample imbalance with a focal loss function. It has achieved good performance in both offline and online data. Wang Z. et al. (2023) combined techniques such as Mixup, stochastic weight averaging, label smoothing, and focal loss during the training of deep learning methods so as to improve the performance of models such as EEGNet in the cross-subject P300 classification task. The single-scale convolution used by these methods may not be able to comprehensively extract the temporal and spatial features of P300. Employing multi-scale convolutional kernels in CNNs enables a more extensive capture of receptive field features within EEG.This capability has the potential to enhance the variety and richness of the extracted feature information, thereby potentially boosting the performance of EEG classification tasks. For example, Altuwaijri et al. (2022) proposed a multi-branch CNN model incorporating an attention module to facilitate effective EEG decoding for motor imagery tasks. Tao et al. (2023) proposed an attention-based convolutional recurrent neural network specifically designed for EEG-based emotion recognition. Additionally, Lan et al. (2021) introduced a multi-attention convolutional recurrent model (MACRO), which is reported to more efficiently extract spatio-temporal features from P300. While deep learning-based EEG classification techniques have demonstrated significant advancements, the comprehensive utilization of spatio-temporal information within EEG data remains a substantial challenge. For instance, prevailing methods may inadequately address the extraction of fine-grained features. Such an oversight can result in the omission of subtle time and space variations within the EEG which contain extensive information across time and space domains. The development of more effective methods for capturing this intricate data is essential for achieving more accurate EEG classification outcomes.

The pyramid squeeze attention (PSA) module has proven effective in extracting multi-scale features from images (Jiang et al., 2022; Yan et al., 2022). As the P300 exhibits a significant positive wave peak on EEG images that appears ~300 ms after stimulation, there is a current need to extract as many of these features as possible. Although the PSA module has not yet been used for EEG classification tasks, its application to P300 feature extraction has the potential to yield finer-grained information. Therefore, the impact of the PSA module on the performance of CNN feature extraction and classification deserves in-depth study.

This study introduces PSAEEGNet, a novel framework that integrates attentional mechanisms within multiscale convolutional neural networks to address the classification of single-trial EEG in RSVP tasks. Comparative analysis reveals that PSAEEGNet surpasses the performance of several existing models, including hierarchical discriminant component analysis (HDCA) (Marathe et al., 2013), shallow convolutional neural network (ShallowConvNet), deep convolutional neural network (DeepConvNet) (Schirrmeister et al., 2017), and EEGNet (Lawhern et al., 2018), in terms of classification accuracy. Notably, the PSAEEGNet achieves marked effectiveness in discriminating P300.

## 2 Materials and methods

### 2.1 Data and data preprocessing

This study uses the RSVP-based brain-computer interface benchmark dataset from Tsinghua University (Zhang et al., 2020), which contains EEG data from 64 healthy subjects with 64 electrode channels, sampled at 250 Hz. EEG were collected while subjects performed a target image detection task. For each subject, two groups of experiments were conducted, with each group containing two blocks. Each block consisted of 40 trials, and each trial was presented with 100 stimulus images randomly at a frequency of 10 Hz. The stimuli consisted of two categories: target images and nontarget images. The target images were presented with a probability of 1%–4%.

In this study, a selection of 62 electrodes (1–32, 34–42, 44–64) were chosen for further processing as Zhang et al. (2020). The EEG data were then filtered using a Butterworth filter with a bandwidth of 2–30 Hz, following the methodology of Lan et al. (2021). Subsequently, a segment spanning 0–1,000 ms was extracted post the onset of each image stimulus. This segment was further divided into data comprising 250 sampling points. As a result, the format of the single-trial EEG data was structured as a matrix of 62 electrodes by 250 sampling points.

### 2.2 PSA module structure

**Figure 1:**
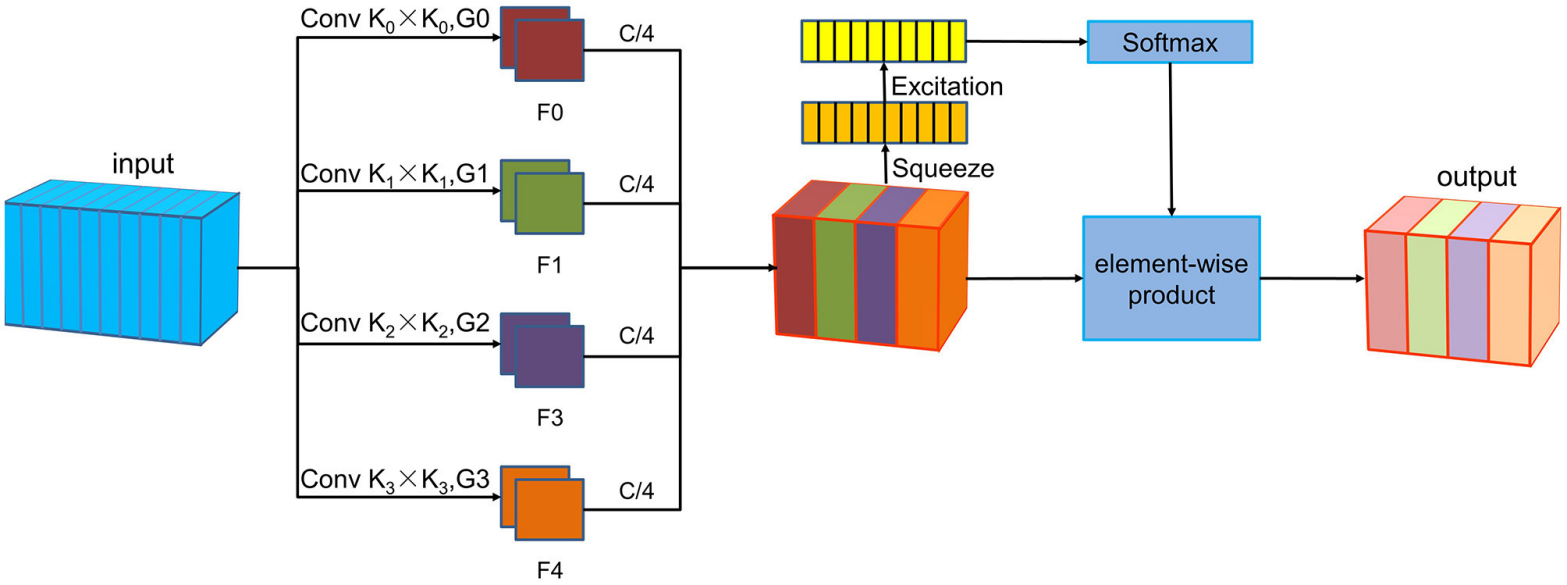
The PSA module architecture. The PSA module includes four steps. First, the Squeeze and Concat module is used for multi-scale feature extraction. Then, the Squeeze and Excitation module processes feature maps of each scale to extract attention information and generate corresponding attention vectors. The Softmax function is then used to normalize these attention vectors. Finally, the normalized weights are multiplied element-wise with the corresponding feature maps.

The PSA module is a lightweight and efficient attention method (Zhang et al., 2023), the implementation of PSA module consists of the following four steps, as shown in Figure 1. The PSA module commences with multi-scale feature extraction using the Squeeze and Concat module. Next, the Squeeze and Excitation block (Hu et al., 2018) processes the feature maps at various scales to extract attention information and produce corresponding attention vectors. These attention vectors are then normalized using the Softmax function. Subsequently, the normalized weights undergo element-wise multiplication with the corresponding feature maps to produce enriched feature maps with comprehensive multi-scale information. In this study, the PSA module enhances feature extraction at a more granular level, which allows for the extraction of more detailed information from the P300, facilitating improved target recognition in brain-computer interface systems.

### 2.3 PSAEEGNet architecture

**Figure 2:**
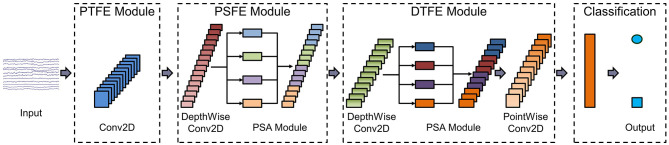
The PSAEEGNet architecture. The PSAEEGNet network structure consists of four simple modules. The first module is the primary temporal feature extraction (PTFE) module, which is mainly used for preliminary extraction and dimensionality reduction of temporal features; the second module is the primary spatial feature extraction (PSFE) module, which is used for preliminary multi-scale extraction of spatial features; the third module is the deep temporal feature extraction (DTFE) module, which carries out multi-scale temporal feature extraction based on the primary temporal feature extraction module; and the fourth module is the classification module, which is used by the fully-connected layer to accomplish the task of binary classification of P300.

This study introduces a CNN model named PSAEEGNet, which incorporates PSA modules. The PSAEEGNet is composed of four key modules: two are dedicated to temporal feature extraction, one to spatial feature extraction, and the final one to classification. The general architecture of the model is illustrated in Figure 2. Table 1 provides detailed information on the main parameter settings used in the model.

**Table 1:** The PSAEEGNet architecture.

**Module**	**Layer**	**Filters**	**Size**	**Output**	**Option**
1	Input	–	–	(6, 250)	–
	Reshape	–	–	(1, 62, 250)	–
	Con2D	8	(1, 125)	(8, 62, 250)	–
	BatchNorm	–	–	(8, 62, 250)	–
2	DepthwiseConv2D	16	(62, 1)	(16, 1, 250)	–
	BatchNorm	–	–	(16, 1, 250)	–
	Activation	–	–	(16, 1, 250)	ELU
	PSA	16	(3, 5, 7, 9)	(16, 1, 250)	–
	BatchNorm	–	–	(16, 1, 250)	–
	Activation	–	–	(16, 1, 250)	ELU
	AveragePooling2D	–	(1, 8)	(16, 1, 31)	-
	Dropout	–	–	(16, 1, 31)	*p* = 0.5
3	DepthwiseConv2D	16	(1, 16)	(16, 1, 31)	–
	PSA	16	(1, 3, 5, 7)	(16, 1, 31)	–
	PointwiseConv2D	16	(1, 1)	(16, 1, 31)	–
	BatchNorm	– –	–	(16, 1, 31)	–
	Activation	–	–	(16, 1, 31)	ELU
	AveragePooling2D	–	(1, 8)	(16, 1, 4)	–
	Dropout	–	–	(16, 1, 4)	*p* = 0.5
4	Reshape	–	–	64	View
	Liner	–	–	2	–
	Activation	–	–	2	Softmax

The first module is the primary temporal feature extraction (PTFE) module, which is mainly used for initial extraction of temporal features and dimensionality reduction, comprising a reshaping layer, a two-dimensional convolutional layer (Conv2D), and a batch normalization (BN) layer (Ioffe and Szegedy, 2015). The reshaping layer transforms the EEG data matrix to match the input format required by the Conv2D layer. Subsequently, the Conv2D layer applies temporal convolution to the processed EEG data. The dimensions of the time-convolution kernel are set at (1, 125). The resulting output feature map has dimensions of eight filters by 62 electrodes and 250 sampling points. A BN layer is also utilized to mitigate distributional shifts (Liu et al., 2018).

The second module is the primary spatial feature extraction (PSFE) module, which is dedicated to the initial extraction of spatial features, incorporating a deep convolutional layer, a PSA module, a BN layer, and a pooling layer. The process begins with the input undergoing convolution via a DepthwiseConv2D layer with a kernel size of (62, 1), subsequently activated through a BN layer and an exponential linear unit (ELU). Following this, the PSA module, armed with an array of convolution kernels sized (3, 5, 7, 9), orchestrates the concurrent convolution of the input, thereby gleaning multi-scale spatial features from the EEG. A subsequent BN layer and ELU activation further refine the features. Finally, a global average pooling layer is utilized to diminish the dimensionality and curtail the likelihood of overfitting.

The third module is the deep temporal feature extraction (DTFE) module, the SeparableConv2D and PSA modules are employed to enhance the extraction of temporal features. The Separable Convolution layer, which comprises DepthwiseConv2D followed by PointwiseConv2D, serves to decrease the number of parameters by partitioning the operations of standard convolution (Lawhern et al., 2018). In the process delineated in this study, each channel undergoes initial convolution individually via DepthwiseConv2D [with a kernel size of (1, 16)]. Subsequently, the PSA module [employing kernels of sizes (1, 3, 5, 7)] extracts multi-scale temporal feature information from the EEG data of each channel, and PointwiseConv2D is then utilized to integrate the inter-channel information. Mirroring the approach in PSFE module, a BN layer and the ELU function are applied following the separable convolutional layer, and global average pooling is subsequently implemented.

In the final classification module, a fully connected layer endowed with a softmax activation function acts as the classifier within the architecture. This layer contains precisely two units, reflecting the binary classification of the EEG data categories. The feature vectors delineated by preceding layers are directly relayed to the fully connected layer, whereupon the model computes the probabilistic decision scores for the target and non-target classes.

Furthermore, Dropout is implemented following modules PSFE and DTFE to expedite the training process and impose regularization on the model (Srivastava et al., 2014; Tompson et al., 2015)

### 2.4 Training settings and implementations

The CNN proposed in this study is fine-tuned using the Adam optimizer, learning rate set to 0.001, adhering to the hyperparameter specifications suggested by Kingma and Ba (2014). Cross-entropy is utilized as the loss function of choice. To counteract the issue of data imbalance, class weights have been integrated within the loss computation. The learning rate is halved if no decrement in validation loss is observed after five consecutive epochs. Additionally, an early stopping protocol is instituted to prevent overfitting and to minimize training duration; training is halted if a reduction in validation loss does not occur within 20 epochs. The batch size for the training process is set at 64.

The deep learning architectures presented in this study were constructed using the PyTorch framework and trained on an NVIDIA RTX 3090 GPU, with the support of CUDA 11.7 and cuDNN v7.6. Consistency in training configurations has been upheld for all the models included in this research.

### 2.5 Evaluation index

The performance of the PSAEEGNet was evaluated using a suite of metrics: accuracy (ACC), true positive rate (TPR), false positive rate (FPR), F1-score, and the area under the receiver operating characteristic curve (AUC) (Cho and Jang, 2020). These metrics can be shown in the following way (Equations 1–5):


(1) $$\begin{array}{l} Accuracy=\frac{TP+TN}{TP+TN+FP+FN} \end{array}$$

(2) $$\begin{array}{l} TPR=\frac{TP}{TP+FN} \end{array}$$

(3) $$\begin{array}{l} FPR=\frac{FP}{FP+TN} \end{array}$$

(4) $$\begin{array}{l} F1-score=\frac{2\times TP}{2\times TP+FP+FN} \end{array}$$

(5) $$\begin{array}{l} AUC=\frac{1}{P\times N}\overset{P}{\underset{i=1}{\sum }}\overset{N}{\underset{j=1}{\sum }}I\left(p_{i}>p_{j}\right) \end{array}$$

## 3 Results

### 3.1 Role of PSA module

This section focuses on the experimental assessment of how varying numbers of PSA modules and convolutional kernels within these modules affect the feature extraction efficacy and subsequent classification accuracy of CNN. The empirical research conducted in this segment is instrumental in iteratively refining and ultimately defining the architecture of the PSAEEGNet model. Stratified five-fold Cross-Validation was used to test all models for each subject. This validates the performance of all models and algorithms in small sample training situations (Zang et al., 2021).

#### 3.1.1 Experiments with different number of PSA module combinations

To systematically evaluate the efficacy of PSA modules, a series of CNN architectures were meticulously designed and implemented for experimental investigation. Each architecture featured distinct combinations of PSA module counts, with the foundational design principles drawn from the original PSA module as described by Zhang et al. (2023). In these configurations, the PSA module convolutional kernel is (3, 5, 7, 9), collectively referred to as “A modules.” On the other hand, architectures that did not incorporate any PSA modules were identified by the label 0. Subsequently, the classification performance of the CNNs is systematically compared across various combinations of PSA module counts.

**Figure 3:**
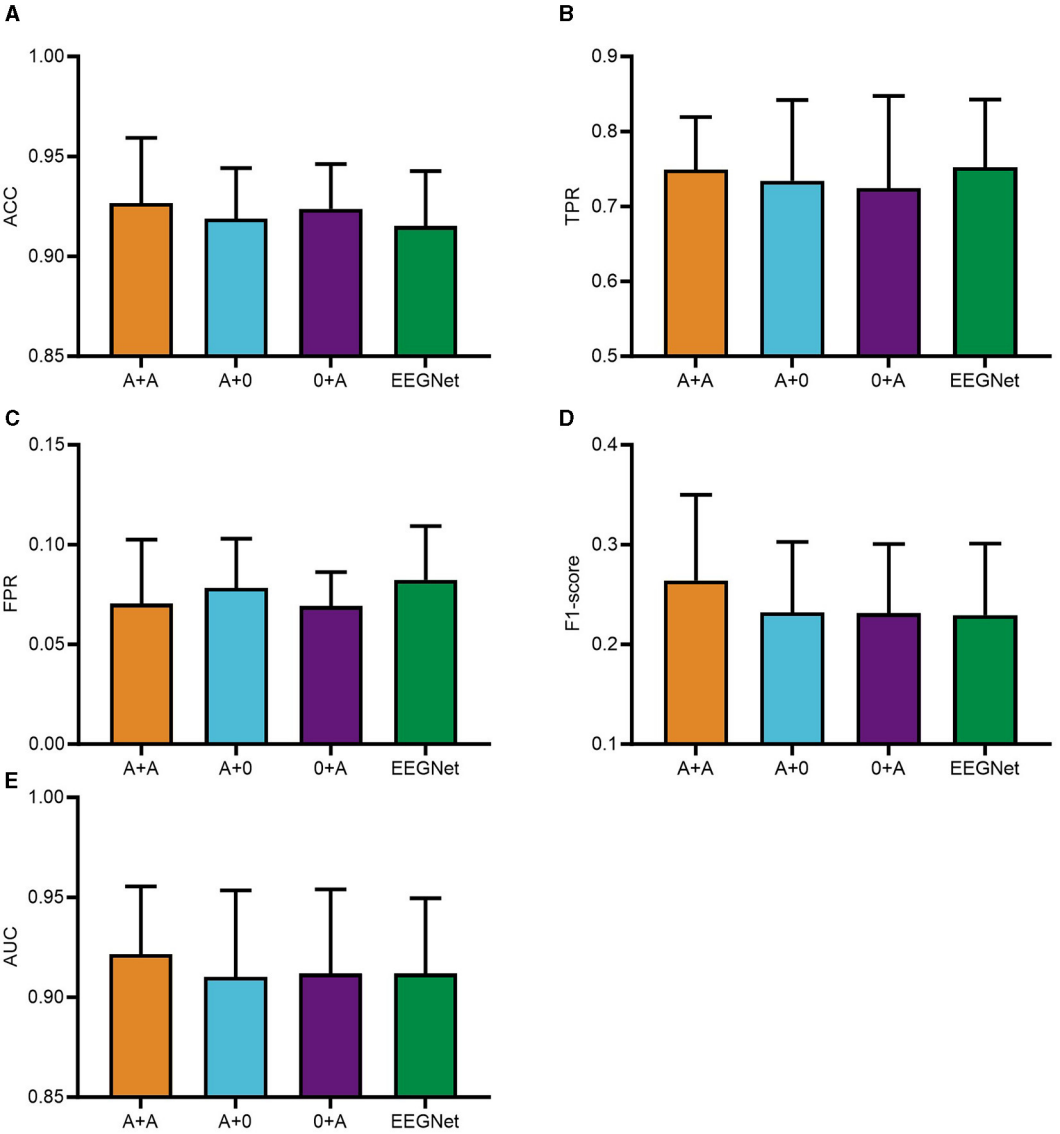
PSAEEGNet classification results for different combinations of number of PSA modules. In the figure, A+A refers to the model with PSA modules added to PSFE module and DTFE module of the network structure; A+0 is the model with PSA modules added to PSFE module of the network structure only; and 0+A is the model with PSA modules added to DTFE module of the network structure only. EEGNet is the blank control group with no PSA modules added. Panels **(A–E)** show the performance of A+A, A+0, 0+A, and EEGNet in the metrics ACC, TPR, FPR, F1-score, and AUC, respectively, and the error bars represent the standard deviation.

The integration of the PSA module into PTFE module of the CNN was found to significantly impair computational efficiency. As a result, the PSA modules were placed after the feature extraction phases in modules PSFE and DTFE of the CNN framework. The experimental phase of this study utilized a consecutive series of data from five subjects, selected in their ordinal sequence within the dataset. Subsequently, a comparison of the classification performance was conducted for the four distinct CNN configurations: A+A, where PSA modules were integrated into both PSFE module and DTFE module; A+0, where a single PSA module was added to PSFE module only; 0+A, where the PSA module was incorporated solely into DTFE module; and where the EEGNET module was blank control module. The comparative outcomes are presented graphically in Figure 3.

Figure 3 illustrates the performance of the A+A group, A+0 group, 0+A group, and the baseline EEGNet across various metrics including ACC, TPR, FPR, F1-score, and AUC. To begin with, regarding ACC, the A+A model demonstrates superior performance with an accuracy of 0.9267; the 0+A model achieving an accuracy of 0.9238; the A+0 model records an accuracy of 0.9189; whereas EEGNet reports an accuracy of 0.9152. As for TPR, the A+A model exhibits strong recognition capability, with a TPR of 0.7491; the TPRs of the A+0 and 0+A models are 0.7342 and 0.7242, respectively, whereas EEGNet displays a TPR of 0.7522. Concerning FPR, the differences between the four experimental models are relatively minor, with the 0+A model attaining the lowest FPR of 0.0692, closely followed by the A+A model at 0.0705, then the A+0 model with an FPR of 0.0782, and EEGNet with an FPR of 0.0823. With respect to the F1-score, all four models display modest scores, yet the A+A model outperforms others with a score of 0.264, surpassing the scores of 0.2319 (A+0), 0.2311 (0+A), and 0.2293 (EEGNet). Lastly, when examining AUC values, the A+A model achieves the highest AUC at 0.9216, indicative of its overall better performance. Following closely is EEGNet with an AUC of 0.9119, while the 0+A model registers 0.9118, and the A+0 model records an AUC of 0.9102.

Figure 3 illustrates that the A+A group surpassed the remaining groups concerning ACC, FPR, F1-score, and AUC metrics. These results underscore the effectiveness and superiority of the two PSA modules. Consequently, the study adopted a CNN model that integrates two PSA modules for further analysis.

#### 3.1.2 Experiments on convolutional kernel parameters for PSA modules

In CNNs, selecting appropriate convolutional kernel parameters is pivotal for enhancing the feature learning capacity of the module. The size of these kernels dictates the scale at which the model discerns and assimilates features. A pyramid multi-scale approach is employed in the PSA module, incorporating concurrent use of variously sized convolutional kernels. This strategic adoption allows for the capture of diverse scale features, thereby significantly boosting the feature extraction capabilities of CNN.

**Figure 4:**
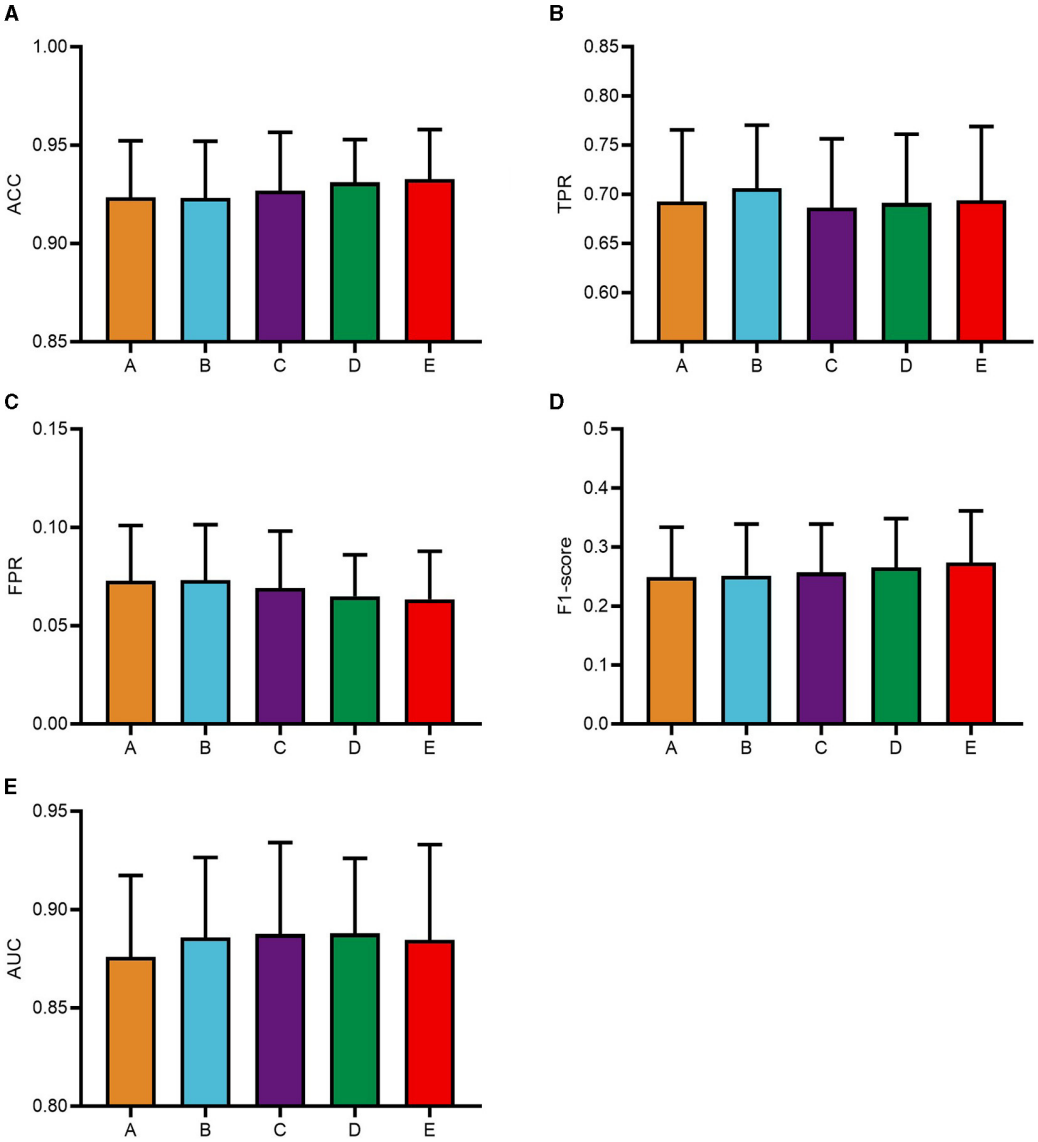
Classification results of PSAEEGNet with different convolutional kernel configurations. Based on the A+A model, only the convolutional kernel of the second PSA module of the model was modified and categorized into five groups in this study, and A, B, C, D, and E on the horizontal coordinates of the figure are the models using the convolutional kernel (3, 5, 7, 9), (1, 3, 5, 7), (5, 7, 9, 11), (7, 9, 11, 13), and (9, 11, 13, 15), respectively. Panels **(A–E)** show the performance of the five models A, B, C, D, and E for the metrics ACC, TPR, FPR, F1-score, and AUC, respectively, and the error bars represent the standard deviation.

To systematically analyze the influence of the pyramid multi-scale convolutional kernel sizes within the PSA module on CNN classification performance and to identify optimal convolutional kernel parameters, this study focuses on five distinct configurations. The experimental phase of this study utilized a consecutive series of data from five subjects, selected in their ordinal sequence within the dataset. Based on the A+A combination, this study solely modifies convolutional kernel parameters of the second PSA module into five categories: module A with kernels (3, 5, 7, 9), module B featuring (1, 3, 5, 7), module C utilizing (5, 7, 9, 11), module D adopting (7, 9, 11, 13), and module E employing (9, 11, 13, 15). The module A+A combination is named as group A, A+B as group B, and so on. The classification results of these five groups of models are shown in Figure 4.

The classification metrics for each group were as follows: group A achieved an ACC of 0.9234, group B scored 0.9232, group C attained 0.9269, group D recorded 0.9311, and group E reached the highest ACC at 0.9326. Regarding TPR, the respective values were 0.6928 (group A), 0.7061 (group B), 0.6865 (group C), 0.6912 (group D), and 0.694 (group E). For FPR, the outcomes were 0.07276 (group A), 0.07314 (group B), 0.06908 (group C), 0.0649 (group D), and 0.06338 (group E). In terms of F1-scores, the figures stood at 0.2489, 0.2515, 0.2566, 0.2655, and 0.2732 for groups A through E respectively. Lastly, when assessing the AUC, the results showed 0.876 for group A, 0.8857 for group B, 0.8875 for group C, 0.8879 for group D, and 0.8845 for group E.

The data depicted in Figure 4 reveal that group B outperforms the other groups in terms of TPR, suggesting that it is superior in its ability to accurately identify positive instances, which is particularly important for applications that prioritize the accurate identification of positive samples, such as medical diagnosis and target detection. And there is more to model selection than purely numerical considerations. Notably, model B has the smallest multi-scale convolutional kernel, which reduces computational complexity. Taken together, model B, with its excellent TPR and low computational burden, is a suitable choice for subsequent experiments. Therefore, the convolutional kernel combination of module A+B is selected as the optimal configuration for the CNN model PSAEEGNet in this study.

### 3.2 Classification performance

**Figure 5:**
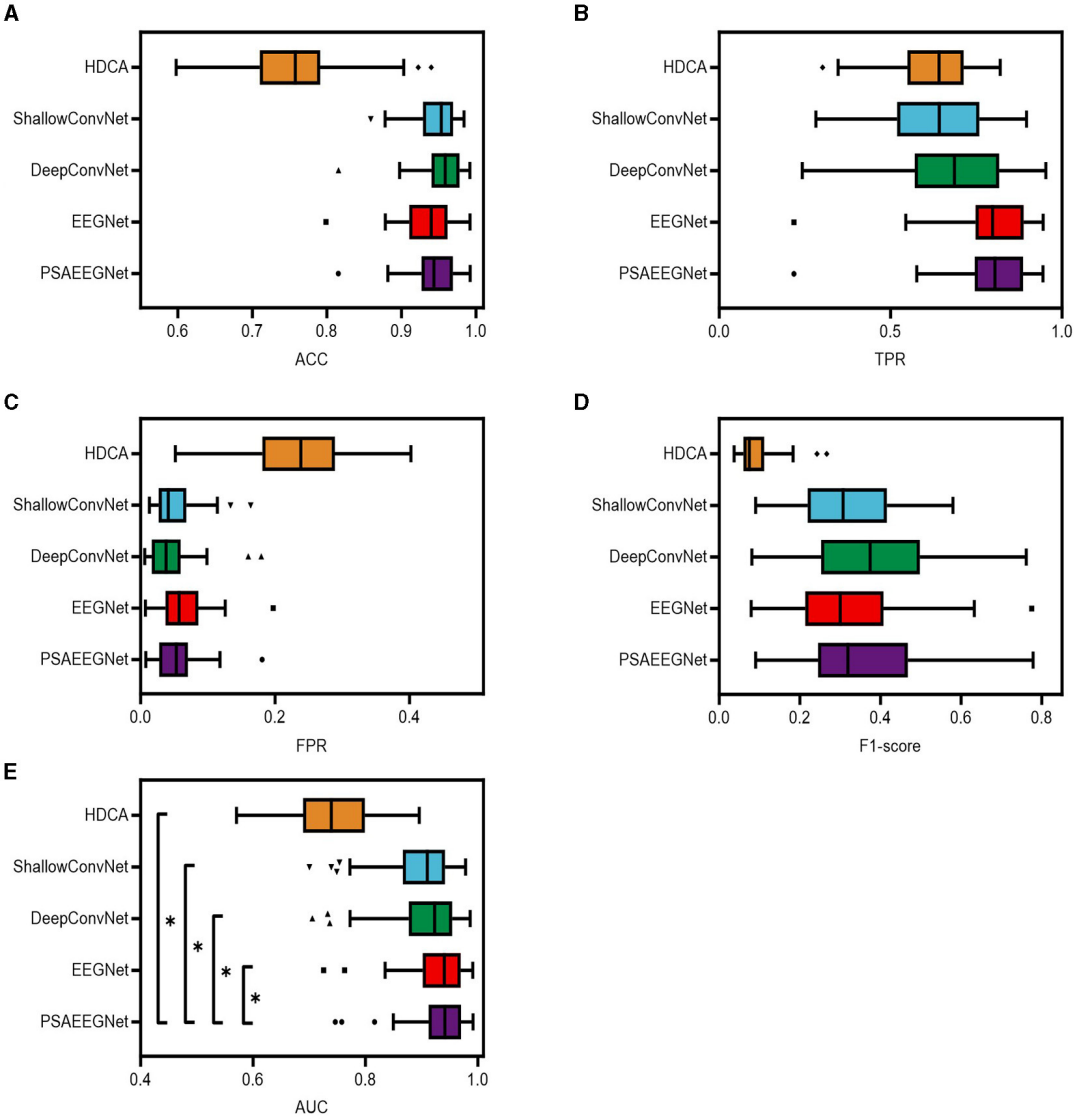
Classification results of different methods. In this study, the five methods of PSAEEGNet, HDCA, EEGNet, ShallowConvNet and DeepConvNet are referred to as groups P, H, E, S, and D. Panels **(A–E)** show the performance of groups P, H, E, S, and D in the metrics ACC, TPR, FPR, F1 score, and AUC, and the error bars represent the standard deviation, and * represents *p* < 0.05.

To assess the single-trial EEG classification capabilities of PSAEEGNet under the RSVP task, a series of experiments were performed using data from all 64 participants in the dataset. The performance of PSAEEGNet was evaluated by contrasting it against conventional machine learning algorithms and several deep learning models, notably HDCA, EEGNet, ShallowConvNet, and DeepConvNet. Figure 5 visually represents the comparative classification performance outcomes for each method.

Figure 5 presents the performance metrics for PSAEEGNet (group P), HDCA (group H), EEGNet (group E), ShallowConvNet (group S), and DeepConvNet (group D) across several dimensions: ACC, TPR, FPR, F1-score, and AUC. The specific metric values are as follows: group P achieved an ACC of 0.944, a TPR of 0.7969, an FPR of 0.05321, an F1-score of 0.3644, and an AUC value of 0.9349. For group E, the corresponding figures were 0.935 for ACC, 0.7922 for TPR, 0.06252 for FPR, 0.3236 for the F1-score, and an AUC of 0.9302. In group D, the five indicators were recorded as 0.9551 for ACC, 0.6792 for TPR, 0.04209 for FPR, 0.382 for the F1-score, and an AUC of 0.9074. group S demonstrated performance with ACC at 0.9476, TPR at 0.6286, FPR at 0.04926, F1-score at 0.3179, and an AUC value of 0.8977. Lastly, group H exhibited weaker results in comparison, with respective scores of 0.7625 for ACC, 0.6267 for TPR, 0.2325 for FPR, 0.0911 for the F1-score, and an AUC of 0.7431.

As depicted in Figure 5, PSAEEGNet exhibits superior performance relative to other methods for the TPR and AUC metrics. A one-way repeated measures analysis of variance (ANOVA) statistical test was employed to scrutinize the existence of significant differences in performance among the various methods. The results from this evaluation indicated that the AUC of PSAEEGNet exhibited significantly superior performance compared to all other methods under consideration (*p* < 0.05). This suggests that PSAEEGNet possesses heightened sensitivity and greater discriminative capacity, enabling it to more effectively identify positive samples and accurately distinguish between positive and negative instances.

## 4 Discussion

### 4.1 Effect of different number of PSA module combinations on CNN classification performance

This study evaluates the classification performance of four CNN models featuring distinct PSA module combinations to identify the optimal number of such modules. The comparative analysis involves four specific configurations: A+A, A+0, 0+A, and EEGNET.

In the context of a CNN where only one PSA module is employed, there exists the possibility to integrate PSA modules into modules PTFE, PSFE, or DTFE to create new network architectures. Experimental evidence suggests that positioning a PSA module in PTFE module significantly impairs computational efficiency, rendering such models impractical. Thus, this configuration is not pursued. When the PSA module is utilized solely in either PSFE module or DTFE module, the constructed A+0, 0+A models exhibit average TPRs of 0.7342 and 0.7242, respectively, with the A+0 group marginally outperforming the 0+A group. The differences in other metrics are minimal, suggesting that separate usage of the PSA module during the temporal and spatial information processing stages of the CNN model can indeed have an impact on classification performance. However, when the PSA module is incorporated into both PSFE module and DTFE module, the mean values of ACC, F1-score, and AUC for the A+A group exceed those of the A+0 and 0+A groups. These results demonstrate the superiority and effectiveness of using dual PSA modules within the CNN model.

The temporal dimension is condensed through a conventional convolutional layer that segregates distinct temporal segments. This is succeeded by a deep convolutional process for the extraction of spatial characteristics, paired with the integration of a PSA module to facilitate the amalgamation of multi-scale spatial domain information. Subsequently, the PSA module is reapplied during the depthwise separable convolution stage to enable the extraction of multi-scale temporal domain information. Such an architectural configuration empowers the group A+A to more effectively discern critical features, thereby enhancing the robustness and accuracy of the model.

### 4.2 Effect of PSA modules with different convolutional kernels on CNN classification performance

A myriad of hyper-parameter settings critically impact the classification efficacy of CNNs. In this research, the experimental focus was on the permutations of multi-scale convolutional kernel sizes within the PSA module.

This study focuses on the A+A configuration and selectively modifies the convolutional kernel parameters of the second PSA module, which is partitioned into five subgroups: A, B, C, D, and E. The kernel sizes are as follows: module A employs (3, 5, 7, 9), module B employs (1, 3, 5, 7), module C employs (5, 7, 9, 11), module D employs (7, 9, 11, 13), and module E employs (9, 11, 13, 15). Upon conducting a one-way ANOVA analysis, no statistically significant difference was found in the classification performance among these five groups, suggesting that the size of the convolutional kernels has a marginal impact on the classification capabilities of CNN. However, the average TPRs across the models were 0.6928, 0.7061, 0.6865, 0.6912, and 0.694 for groups A through E, respectively. Notably, group B exhibited the highest mean TPR. Given the limited sample size and inherent class imbalance within the dataset, it is justifiable to select the configuration with the higher average TPR, thus making the PSAEEGNet of group B the final model.

### 4.3 Comparative performance analysis with other methods

In this study, PSAEEGNet was utilized to process a single-trial EEG dataset informed by the RSVP paradigm, and its performance was compared against a range of established classification methodologies, including HDCA, EEGNet, DeepConvNet, and ShallowConvNet. Analytical results revealed that HDCA underperformed in detecting ERPs, a deficiency principally due to the analysis of traditional EEG reliance on manually curated features, which fail to fully exploit the salient information contained within EEG (Jiao et al., 2018). When benchmarked against other sophisticated deep learning techniques, PSAEEGNet achieved a mean TPR of 0.7969, registering a modest improvement of 0.47% over EEGNet, and markedly outperforming ShallowConvNet and DeepConvNet by 16.83 and 11.77%, respectively. In terms of the AUC metric, PSAEEGNet attained a mean value of 0.9349, which constitutes a 0.47% increment compared to EEGNet and substantial margins of 3.72 and 2.75% over ShallowConvNet and DeepConvNet, respectively. In this study, a one-way ANOVA was employed to determine if there exists a statistically significant difference in performance between PSAEEGNet and other classification techniques. The analysis showed that PSAEEGNet significantly outperformed all other methods in terms of AUC (*p* < 0.05). Notably, PSAEEGNet significantly improves the AUC while sustaining a high TPR, an attribute of paramount importance for real-world applications. Furthermore, it is remarkable that PSAEEGNet maintains robust F1-scores and ACC even with constrained sample sizes, underscoring its ability to perform precise classification and prediction in the context of limited data availability. This attribute presents a viable solution in scenarios characterized by data paucity.

Consequently, the findings of this study permit the conclusion that PSAEEGNet exhibits robust performance across five critical metrics. PSAEEGNet effectively accomplishes single-trial EEG classification within the RSVP task, underscoring the potential and practicality of the method, especially in contexts characterized by data imbalance.

### 4.4 Limitations and future perspectives

The incorporation of multiple PSA modules into the CNN architecture inherently escalates its complexity, which subsequently leads to an increased parameter count and may slightly affect the computational speed of the module. Nevertheless, PSAEEGNet exhibits remarkable effectiveness in classifying EEG, skillfully differentiating between P300 and non-P300 by leveraging the pyramid multi-scale convolution and the attention mechanisms provided. This results in a notable superiority of PSAEEGNet over comparative methods in terms of classification accuracy and discriminative power. The use of more optimization methods and better hardware to improve the computational efficiency of this research method is considered for future research in real-time BCI applications. Deep learning methods have been applied in areas such as motor imagery (Zhu et al., 2022) and cross-subject P300 classification (Wang Z. et al., 2023), so further research based on the methods of this study can be generalized to more application areas and advance the development of BCI systems.

## 5 Conclusion

EEG based on the RSVP paradigm can be used to discover image targets, however, EEG are characterized by non-smoothness and low signal-to-noise ratios, and in particular, there is a greater difficulty in the recognition of image targets using a single-trial unbalanced EEG. To this end, this study proposes a convolutional neural network incorporating a pyramid squeeze attention module, which is novel in that it incorporates an attention mechanism that adaptively extracts the attention of feature maps at different scales, and integrates multiple PSA modules and deep convolutional layer, which can efficiently extract the temporal and spatial domain information of EEG. On this basis, a pyramid multi-scale structure of convolutional kernels is used for parallel processing, and features in EEG can be extracted at a finer granularity level by multiple convolutional kernels, which can improve the recognition accuracy and performance of image targets based on EEG. Comprehensive experiments on RSVP-based single-trial EEG dataset show that PSAEEGNet exhibits higher average TPR (0.7949) and AUC (0.9341, *p* < 0.05) in terms of single-trial EEG classification performance under the RSVP task compared to existing algorithms. In the future, the proposed algorithm will be used in brain-computer interface systems, which can significantly improve the efficiency of image target recognition.

## Data availability statement

Publicly available datasets were analyzed in this study. This data can be found at: http://bci.med.tsinghua.edu.cn/download.html.

## Author contributions

ZY: Conceptualization, Investigation, Writing – original draft, Writing – review & editing. QZho: Data curation, Formal analysis, Writing – review & editing. BW: Methodology, Validation, Writing – review & editing. QZha: Methodology, Writing – review & editing. YY: Data curation, Software, Writing – review & editing. YZ: Validation, Writing – review & editing. YG: Writing – review & editing, Supervision. JZ: Project administration, Writing – review & editing, Supervision. CW: Funding acquisition, Writing – review & editing, Supervision.
